# Peptides from American alligator plasma are antimicrobial against multi-drug resistant bacterial pathogens including *Acinetobacter baumannii*

**DOI:** 10.1186/s12866-016-0799-z

**Published:** 2016-08-19

**Authors:** Stephanie M. Barksdale, Evelyn J. Hrifko, Ezra Myung-Chul Chung, Monique. L. van Hoek

**Affiliations:** 1 School of Systems Biology, George Mason University, Manassas, VA USA; 2 College of Science, George Mason University, Manassas, VA USA; 3 National Center of Biodefense and Infectious Diseases, George Mason University, 10920 George Mason Cir, 10920 George Mason Circle, MSN 1H8, Manassas, VA 20110 USA; 4 Present Address: STCube Pharmaceuticals, Inc., 401 Professional Dr. Suite 108, Gaithersburg, MD 20879-3429 USA

**Keywords:** Antimicrobial peptides, *Alligator mississippiensis*, Multi-drug resistant bacteria, *Acinetobacter baumannii*, *Escherichia coli*, *Staphylococcus aureus*

## Abstract

**Background:**

Our group has developed a new process for isolating and identifying novel cationic antimicrobial peptides from small amounts of biological samples. Previously, we identified several active antimicrobial peptides from 100 μl of plasma from *Alligator mississippiensis*. These peptides were found to have in vitro antimicrobial activity against *Pseudomonas aeruginosa* and *Staphylococcus aureus.* In this work, we further characterize three of the novel peptides discovered using this process: Apo5, Apo6, and A1P.

**Results:**

We examined the activity of these peptides against multi-drug resistant strains and clinical isolates of common human pathogens. We investigated their structural characteristics using circular dichroism and tested for membrane disruption and DNA binding. These peptides were found to have strong in vitro activity against multi-drug resistant and clinically isolated strains of *S. aureus, Escherichia coli, P. aeruginosa*, and *Acinetobacter baumannii.* Apo5 and Apo6, peptides derived from alligator apolipoprotein C-1, depolarized the bacterial membrane. A1P, a peptide from the serpin proteinase inhibitor, did not permeabilize membranes. Performing circular dichroism analysis, Apo5 and Apo6 were found to be predominantly helical in SDS and TFE buffer, while A1P has significantly different structures in phosphate buffer, SDS, and TFE. None of these peptides were found to be hemolytic to sheep red blood cells or significantly cytotoxic up to 100 μg/ml after 24 h exposure.

**Conclusions:**

Overall, we suggest that Apo5 and Apo6 have a different mode of action than A1P, and that all three peptides make promising candidates for the treatment of drug-resistant bacteria, such as *A. baumannii.*

## Background

Cationic antimicrobial peptides (CAMPs) are short proteins produced by the innate immune system of virtually all eukaryotic organisms [1]. CAMPs have a variety of structures, such as helical, beta-sheet, or mixed structures [2], and antibacterial mechanisms, including membrane perturbation [3], DNA binding [4], and enzyme interference [5]. There has been strong interest in the discovery and exploration of CAMPs due to rising drug resistance in pathogenic bacteria and the subsequent need for new therapeutics [1]. Traditionally, new CAMPs are identified through either bioinformatics or fractionation. CAMPS are a diverse group of peptides that can differ by nucleic acid sequence, amino acid sequence, and secondary structure [2], which can complicate the process of locating new CAMPs by analysis of gene and protein databases. Fractionation through HPLC or other methods typically requires large amounts of biological sample, usually a liter or more [6], which can be problematic when working with small or endangered animals. Fractionation and testing can also be challenging due to dilution of samples, as CAMPs can be present in very small amounts [6, 7].

Our group recently developed a novel and powerful process for discovering and identifying new and potentially useful CAMPs from biological samples, called the Bioprospecter process [8]. Our CAMP discovery process is sample-agnostic and requires very small sample volumes for analysis. We have successfully employed this process to discover a large number of new peptides from 100 μl of plasma from the American alligator, *Alligator mississippiensis*; five of these show antibacterial activity against Gram-positive and/or Gram-negative bacteria. Moreover, it is unique in its approach to CAMP identification in that it directly mines the native antimicrobial peptidome through custom-made hydrogel particles whose properties complement the physico-chemical properties of CAMPs [8]. This bioprospecting approach provides us access to the CAMP peptidomes of some of the world’s most remarkable species, to dramatically expand the current CAMP library and potentially unlock the key to overcoming antibiotic resistance via the discovery of new antimicrobial peptides.

Using this tool, we previously described three novel peptides from *A. mississippiensis* (Apo5, Apo6, and A1P) with activity against laboratory strains of *E. coli, P. aeruginosa, S. aureus*, and *Bacillus cereus* [8]. Apo5 and Apo6 are highly related peptides; nested fragments of a purported apolipoprotein. Apolipoproteins have antimicrobial activity against a variety of pathogenic bacteria [9–14] (see Discussion). A1P is the C-terminal fragment of alpha-1-proteinase inhibitor of the serpin family; this protease inhibitor has broad protease inhibiting activity, as well as immunomodulatory effects [15]. In this work, we further characterize the antimicrobial activity of these peptides against bacterial pathogens resistant to multiple antibiotics as well as clinical isolates, including multi-drug resistant *A. baumannii,* which was not previously tested. In addition, we sought to determine the secondary structure of these peptides in order to understand the mechanism by which they exert their antibacterial activity.

## Methods

### Bacteria

*Staphylococcus aureus* ATCC 33592 (MDR) and BAA-1718, *Escherichia coli* ATCC 51659 (MDR) and 4157, *Pseudomonas aeruginosa* ATCC BAA-2110 (MDR), and *Acinetobacter baumannii* ATCC BAA-1794 (MDR) and 9955 were purchased from the American Type Culture Collection (Manassas, VA). *Pseudomonas aeruginosa* strain PAO1 was generously provided by Karin Sauer from Binghamton University (Binghamton, NY). All strains are clinical isolates except for *E. coli* ATCC 4157. Bacteria were grown in Nutrient Broth (Difco 234000) overnight in a shaking incubator (37 °C). Bacteria were aliquoted and frozen at -80 °C and enumerated via serial dilution and plating prior to experimentation.

### Peptides

All peptides were synthesized to order by ChinaPeptides, Inc (Shanghai, China) using Fmoc chemistry. Peptides were provided at >95 % purity, and purity and structure were confirmed with RP-HPLC and ESI-MS.

### Bioinformatics

Physiochemical properties were calculated using the Antimicrobial Peptide Database (APD2) [16]. The percent hydrophobicity is defined as the ratio of hydrophobic residues to total residues. The full-length sequences for the apolipoprotein C-1 containing Apo5 and Apo6 (Accession XP_006276575.1) and for the alpha-1-proteinase containing A1P (Accession XP_006266331.1) were found on the BLAST NCBI database [17]. Ribbon models displaying the full proteins were created using SWISS MODEL [18–20]. The *A. mississippiensis* apolipoprotein C-1 was modeled on the human apolipoprotein C-1 (SMTL id: 1ioj.1.A, Sequence identity = 43.40 %), and the *A. mississippiensis* alpha-1-proteinase was modeled on alpha1-antitrypsin (SMTL id: 3dru.1.A, Sequence identity = 51.41 %) [18–23]. The secondary structure of Apo5, Apo6, and A1P was predicted using I-TASSER [24] and visualized with Chimera [25]. Helical wheel projections and hydrophobic moment were calculated using HeliQuest [26].

### Circular dichroism spectroscopy

Circular dichroism (CD) was performed using a Jasco J-1500 spectropolarimeter. 100 μg/ml of peptide was used in each experiment. Samples were allowed to equilibrate for 3 min prior to data collection at 25 °C in a 1 mm path length cuvette. Spectra were collected from 190 to 260 nm at 0.2-nm intervals, with a data integration time of 4 s and a 1 nm bandwidth. Data presented is an average of four spectra. Peptides were analyzed in 10 mM sodium phosphate buffer (6.12 mM sodium monohydrogen phosphate heptahydrate; 3.92 mM monosodium phosphate anhydrous; pH 7.4), 50 % (v/v) trifluoroethanol (TFE) in phosphate buffer, or 60 mM sodium dodecyl sulfate (SDS) in phosphate buffer. Percent contribution to secondary structure was measured using methods determined by Raussens et al. [27].

### Antimicrobial assays

The antimicrobial MIC activity of the peptides was first determined in cation-adjusted Mueller Hinton Broth (BD 212322) in a 96 well plate following the CLSI protocol. Enumerated bacteria were diluted in broth and 10^5^ CFU was added to each well with varying dilutions of peptide. The plate was incubated for 24 h at 37 °C and then read on a spectrophotometer at OD_600_ nm.

The EC_50_ antibacterial activity of the peptides was determined in 10 mM phosphate buffer using resazurin as an indicator of CFU as previously described and validated by Bishop et al. [8] or by colony counting. The sequences and net charges of the peptides tested are shown in Table 1. In a polypropylene 96 well plate, 10^5^ bacteria were incubated in 10 mM phosphate buffer with various dilutions of peptide (3 h; 37 °C) (1:5). For the resazurin method, cation-adjusted Mueller Hinton Broth (BD 212322, final concentration 2.2 %) and resazurin (final concentration 109 μM) dissolved in PBS were added to each well, and the plate was read kinetically overnight on a Tecan Safire^2^ spectrofluorometer (37 °C; excitation = 540 nm; emission = 590 nm). Percent survival after treatment was then calculated with previously determined growth curves (Table 2). For colony counting, experimental wells were serially diluted after incubation, and 8 μl of each dilution was spotted onto cation-adjusted Mueller Hinton Agar (BD 211438) and allowed to dry. Agar plates were then incubated overnight at 37 °C. After incubation, colonies were counted and percent survival was calculated based on untreated bacteria. GraphPad Prism 6.0 was used to calculate EC_50_ using log concentration vs. percent survival. LL-37, polymyxin B (against Gram-negative bacteria), and vancomycin (against Gram-positive bacteria) are used as controls. LL-37 is a well-studied human CAMP with broad-spectrum antimicrobial activity, polymyxin B is a peptide antibiotic effective against Gram-negative bacteria, and vancomycin is a peptide antibiotic used against Gram-positive bacteria. Each experiment was repeated at least twice, and a representative experiment is shown. 95 % confidence intervals (CI) are reported to indicate the error of each EC_50_ determination.

**Table 1:** Amino acid sequences and molecular weights of selected peptides (identified in [8])

Peptide name	Sequence	Molecular weight	Charge	Hydrophobicity	Length
Apo5 APOC1_64-88_	FSTKTRNWFSEHFKKVKEKLKDTFA	3103.57	+4	32 %	25
Apo6 APOC1_67-88_	KTRNWFSEHFKKVKEKLKDTFA	2766.49	+4	31 %	22
A1P_394-428_	PPPVIKFNRPFLMWIVERDTRSILFMGKIVNPKAP	4106.28	+4	45 %	35
LL-37	LLGDFFRKSKEKIGKEFKRIVQRIKDFLRNLVPRTES	4493.33	+6	35 %	37

**Table 2:** Antimicrobial assay bacterial reduction rate equations used to determine CFU after incubation with peptide

Bacterial strain	Reduction rate equation
*P. aeruginosa* PAO1	log(CFU_*P.aeruginosa*_) = (T_20000_ – 60948) / -5823.3
*E. coli* ATCC 51659	log(CFU_*E. coli*_) = (T_20000_ – 37673) / -4292.9
*S. aureus* ATCC 33592	log(CFU_*S. aureus*_) = (T_20000_ – 59514) / -7912.2
*A. baumannii* ATCC BAA-1794	log(CFU_*A. baumannii*_) = (T_20000_ – 34042) / -4398.1

### Ethidium bromide uptake assay

The ethidium bromide uptake assay was performed as previously detailed [28, 29] with some modifications. *E. coli* ATCC 51659 was grown overnight in cation-adjusted Mueller Hinton Broth in a shaking incubator (37 °C). Bacteria was centrifuged, washed with PBS, and then adjusted to an OD_600_ nm of 0.1 in 10 mM phosphate buffer. 180 μl of bacteria was added to 10 μl ethidium bromide (10 mM final concentration) and 10 μl peptide in various concentrations. The plate was read in a Tecan Safire^2^ spectrofluorometer every 2 min for 30 min (37 °C; excitation = 540 nm; emission = 590 nm). Peak RFU at 50 μg/ml was used in Fig. 5.

### Membrane depolarization study

Membrane depolarization was studied using DiSC_3_(5) as has previously been reported [30]. Depolarization of a membrane can be visualized by a drop in fluorescence. Enumerated frozen bacteria were pelleted and washed twice in 10 mM phosphate buffer and then resuspended to 4x10^7^ CFU/ml in 10 mM phosphate buffer containing 50 μg/ml DiSC_3_(5). 100 μl of this suspension was added to wells of a black 96 well plate. The plate was incubated in a Tecan Safire^2^ spectrofluorometer and monitored until fluorescence leveled off. 100 μl of various concentrations of peptide in 10 mM phosphate buffer was added to each well. Bacteria without peptide and peptide without bacteria served as controls. The plate was immediately returned to the spectrofluorometer. Readings were taken every 15 s for 5 min (excitation = 622 nm; emission = 670 nm). Peak RFU at each concentration was used in Fig. 6.

### Gel shift assay

Non-specific DNA-peptide binding was determined by a gel-retardation experiment as described by Park et al. [4]. Briefly, 200 ng of plasmid DNA (pTrcHis, Invitrogen V36020) was incubated with increasing concentrations of peptides in 20 μl of binding buffer (5 % glycerol, 10 mM Tris–HCl [pH 8.0], 1 mM EDTA, 1 mM DTT, 20 mM KCl and 50 μg/ml BSA) at room temperature for 20 min and subjected to electrophoresis on a 1.0 % agarose gel. DNA bands were visualized by ethidium bromide staining.

### Hemolysis and MTT assays

To measure the hemolytic activity of peptides, 2 % sheep red blood cells were added to various dilutions of peptide reconstituted in PBS in a sterile U-bottom 96 well plate. 2 % RBCs with PBS 1X alone served as the negative control, and 2 % RBC in water as the positive control. The plate was incubated for 1 h at 37 °C and then centrifuged at 1000 rpm for 2 min. The supernatant was transferred to a fresh plate and read at OD_540_ [31].

Cytotoxicity assays were performed using the Vybrant MTT Cell Proliferation Assay Kit (Life Technologies) according to manufacturer’s instructions. Assays were performed using human lung epithelial lung carcinoma line A549 (ATCC CCL-185), which were maintained at a low passage in Dulbecco’s Minimal Essential Media (Life Technologies 11995073) with 10 % heat-inactivated fetal bovine serum and 13 U/ml penicillin-streptomycin. 1–100 μg/ml of peptide was used for each experimental well. Each experiment was performed in triplicate two times. A representative experiment is shown.

### Statistics

Statistical analyses were performed using GraphPad Prism 6.0. To determine statistical significance, the one-way ANOVA with Tukey’s multiple comparisons was performed in all instances.

## Results

### Bioinformatics

**Fig. 1:**
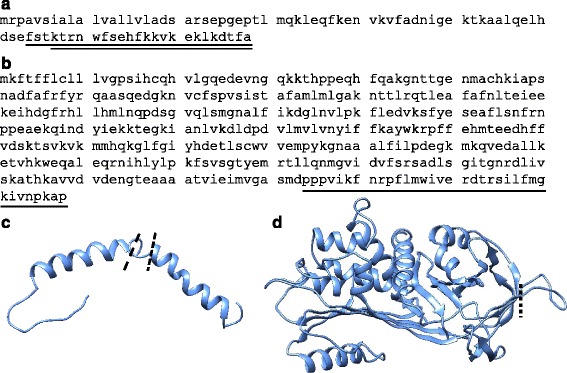
Primary and tertiary structure of parental proteins and peptide placement within. **a** Amino acid sequence of *A. mississippiensis* apolipoprotein C-1 and the Apo5 and Apo6 fragments. **b** Amino acid sequence of *A. mississippiensis* alpha-1-proteinase 2-like protein and the A1P fragment. **c** Ribbon model of apolipoprotein C-1 showing cleavages points of Apo5 (*long dashes*) and Apo6 (*short dashes*). **d** Ribbon model of alpha-1-proteinase showing cleavage point of A1P

The native, plasma-derived peptide sequences from the *de novo* sequencing [8] were used to predict the secondary structure and placement within the parent proteins. Apo5 and Apo6 are both part of the C-terminus of an apolipoprotein, apolipoprotein C-1. Apo5 comprises amino acids (aa) 64-86, while Apo6 is the smaller fragment (aa 67-86, shown in Fig. 1a). Both peptides have a +4 charge and a hydrophobic ratio of just over 30 % as determined using APD2 [16]. Apo5 is cleaved at a Glu-Phe site and Apo6 at a Thr-Lys site. In the full apolipoprotein, both cleavages sites are located in a disordered hinge preceding a C-terminal alpha helix as seen in Fig. 1c.

A1P is also the cleaved C-terminus of its parent protein, a serpin proteinase inhibitor (aa 394-428, shown in Fig. 1b). It carries a +4 charge and a 45 % hydrophobicity ratio. The peptide is cleaved from the parent protein at Asp-Pro site in a disordered region on the exterior of the folded protein, as shown in Fig. 1d. The cleaved peptide itself consists of the two β-sheet regions that run through the interior of the proteinase.

### Secondary structure determination

To determine general secondary structure of Apo5, Apo6, and A1P, CD spectroscopy was used. CD was performed in 10 mM phosphate buffer, 60 mM SDS, and 50 % TFE in phosphate buffer. Many CAMPs, such as LL-37, maintain a random or disordered structure until associated with a membrane or micelle. SDS forms micelles with a negatively charged surface [32], mimicking the bacterial membrane and forcing the CAMP into a more ordered conformation [33, 34].

TFE is used in CD to promote a helical structure [35] and stabilize secondary structure [36].

**Fig. 2:**
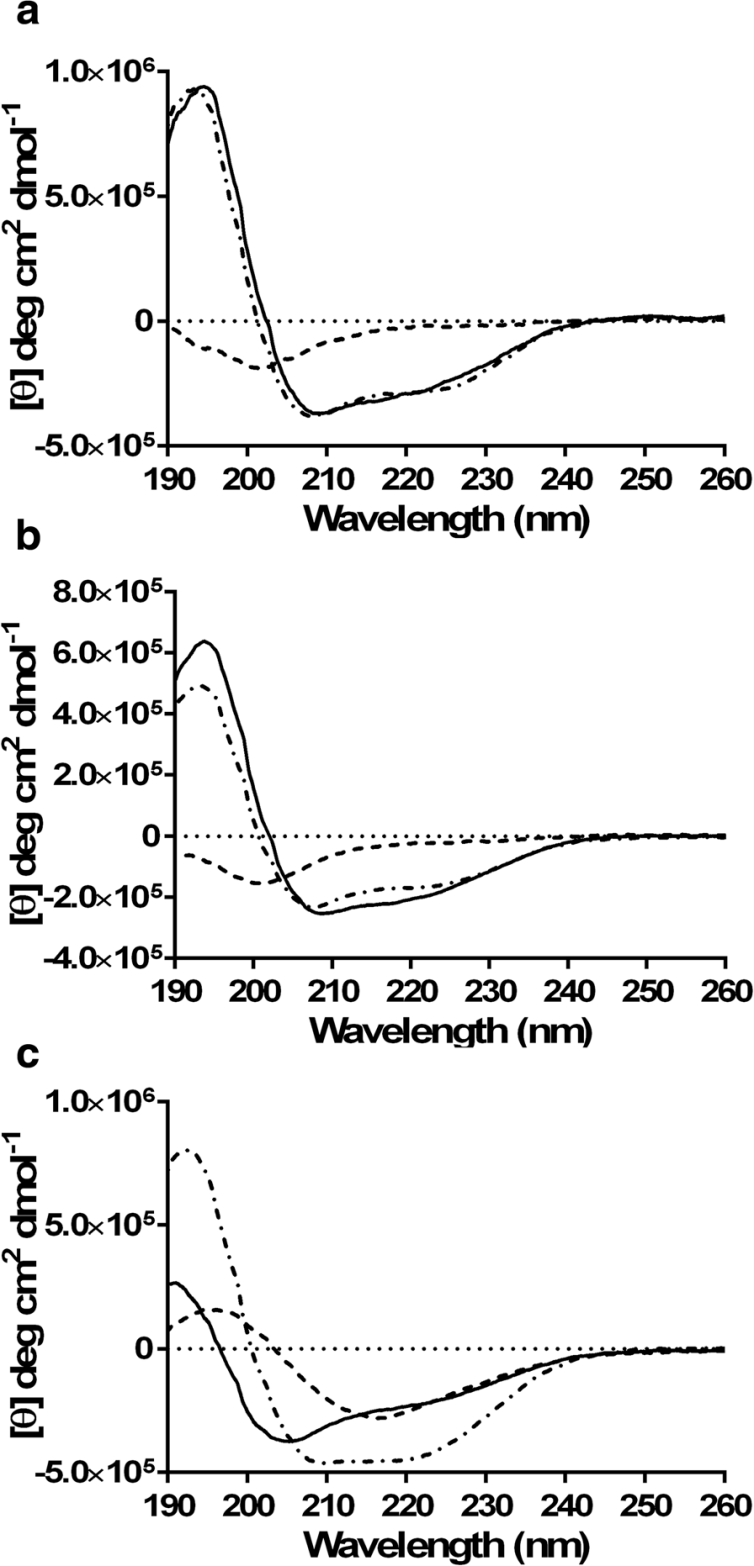
CD spectra of peptides to determine secondary structure. **a** Apo5, **b** Apo6, **c** A1P. All spectra were taken with peptide concentrations of 100 μg/ml in a 1 mm pathway cuvette. Spectra were read in 10 mM phosphate buffer (*long dashes*), 50 % TFE in 10 mM phosphate buffer (*dash dot dash*), and 60 mM SDS in 10 mM phosphate buffer (*solid line*)

**Fig. 3:**
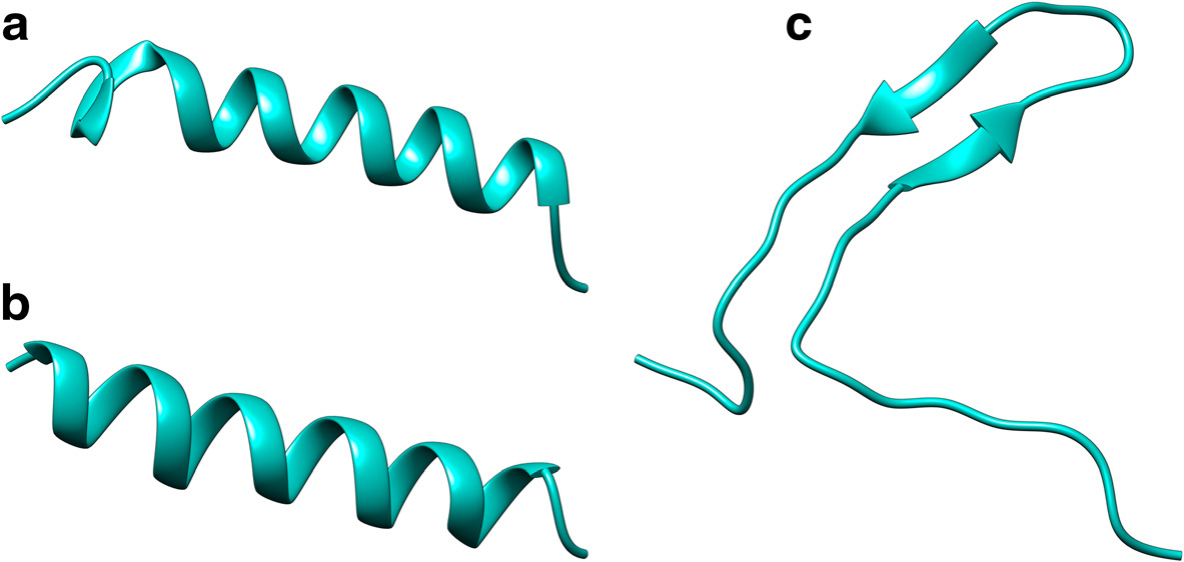
Secondary structure prediction by I-TASSER [24], visualized by Chimera [25]. **a** Apo5, **b** Apo6, **c** A1P

**Fig. 4:**
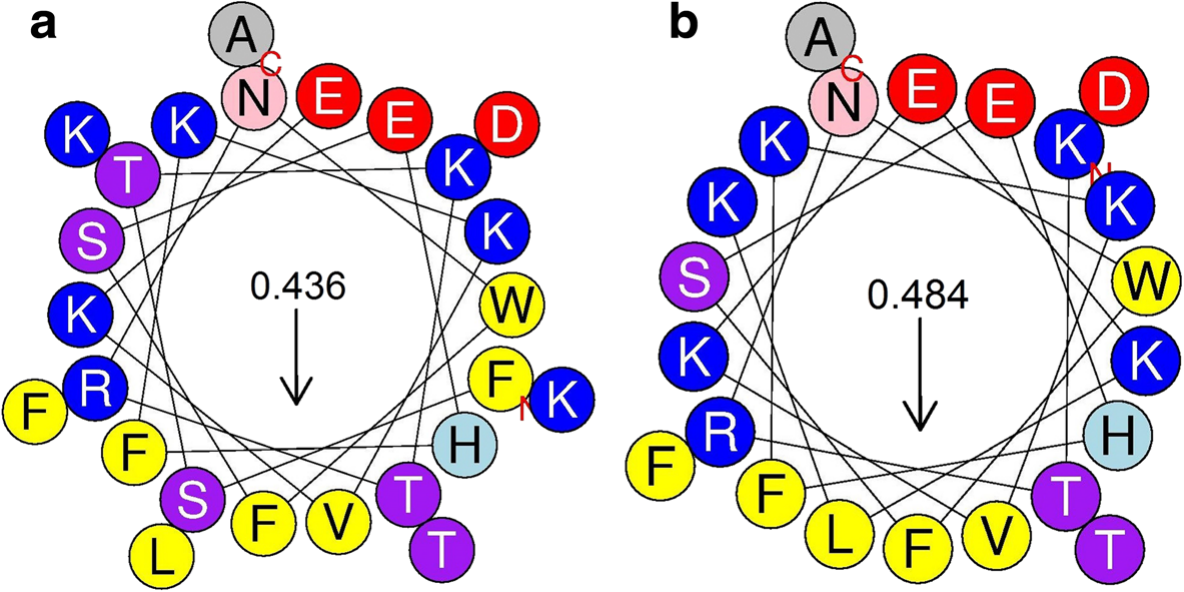
Helical wheels of **a** Apo5 and **b** Apo6, modified from Heliquest

As expected, Apo5 (Fig. 2a) and Apo6 (Fig. 2b) had nearly identical CD spectra. Both peptides have random coil and β-sheet characteristics in 10 mM phosphate buffer, and are primarily α-helical when CD is measured in buffers with SDS and TFE. Interestingly, Apo5, the longer of the two peptides, is calculated to have more α-helical character than Apo6 in both SDS (63.3 % vs. 57.0 %) and TFE (51.0 % vs. 50.3 %), shown in Table 3. When evaluating the α-helical properties of these two peptides by simple intensity at 208 nm and 222 nm, it is notable that the peaks at these wavelengths are more intense for Apo5 than Apo6, though the two Apo peptides maintain 12.5 % turn no matter the buffer used. Apo5 and Apo6 likely have a primarily α-helical structure with some random coil portions. Based on helical wheel projections, it appears that both Apo5 and Apo6 have significant amphipathic character, with several hydrophobic residues on one face and several basic amino acids on the other. The structure of Apo5 and Apo6 were predicted using I-TASSER and the resulting.pdb file was visualized using Chimera, shown in Fig. 3a and b. Consistent with the CD spectra, both are predicted to be α-helical structures. Apo5, the longer peptide, has a longer random coil portion at the N-terminus. This is consistent with the predicted structure of the C-terminal portion of the parental protein, apolipoprotein C-1 (Fig. 1c). Because Apo5 and Apo6 are predicted to helical, helical wheels were produced using Heliquest and modified (Fig. 4). Apo5 and Apo6 have a similar hydrophobic moment (0.436 vs 0.484); Apo6 likely has a slightly stronger hydrophobic moment due to the loss of polar Ser in the hydrophobic face of the helix. In 10 mM phosphate buffer, TFE, and SDS, A1P has significantly different spectra (Fig. 2c).

In 10 mM phosphate buffer, A1P is calculated to have primarily β-sheet contributions (57.9 %), as well as some random coil characteristics (34.7 %), shown in Table 3. By our calculations, A1P also has a negative percentage of contribution from the α-helix, which may be an artifact of the equations used. In TFE, A1P maintains its random coil nature (31.2 %), but also becomes strongly α-helical (38.2 %), with only 16.3 % contribution from the β-sheet. In SDS, A1P is calculated to be nearly equal parts random coil (36.9 %), turn (21.5 %), β-sheet (24.2 %), and α-helix (21.6 %). It has been shown by several groups that TFE can induce and support α-helical structures in peptides [37, 38]. By qualitatively evaluating the spectra, we conclude that only in TFE does A1P have a notable α-helical structure, though this conformation may be interrupted by several proline residues found along the polypeptide chain. It is likely that A1P has a mixed structure that may change dramatically based on environmental factors. The secondary structure of A1P was predicted by I-TASSER and visualized using Chimera. I-TASSER predicts that A1P is primarily random coil with two anti-parallel β-sheet in a hairpin formation (Fig. 3c), which corresponds with the structure of the full alpha-1-proteinase structure.

**Table 3:** Percent secondary structure contribution as calculated by a method described by Raussens et al. [27]

	α-helical	β-sheet	Turn	Random	Sum
Apo5					
Phosphate Buffer	8.9	30.3	12.5	40.6	92.3
TFE	51	4.6	12.5	29.3	97.4
SDS	63.3	-4.8	12.5	25.1	96.1
Apo6					
Phosphate Buffer	9.4	30.5	12.5	41	93.4
TFE	50.3	4.8	12.5	29.7	97.3
SDS	57	0.7	12.5	26.9	97.1
A1P					
Phosphate Buffer	-14.4	57.9	12.4	34.7	90.6
TFE	38.2	16.3	12.5	31.2	98.2
SDS	21.6	24.2	21.5	36.9	104.2

### Antimicrobial activity

Previously, we reported that Apo5 and Apo6 had strong broad-spectrum antimicrobial activity against *E. coli* and *B. cereus*, as well as *S. aureus* and *P. aeruginosa* [8]. It was also found that A1P had greater activity against *S. aureus* than *P. aeruginosa* [8]*,* indicating it may have stronger activity against Gram-positive organisms.

Antibiotic resistance has been increasing steadily for the past several decades, and CAMPs are considered a possible basis for novel antimicrobials. Because of this, these new peptides were tested against clinically isolated and multi-drug resistant strains of *S. aureus*, *E. coli*, *P. aeruginosa* and *A. baumannii*. Numerical and statistical data can be found in Table 4. Sharing a salt-sensitive phenotype with LL-37 [34, 39, 40], these peptides had no effect in MIC experiments as high as 250 μg/ml, so experiments were performed to determine the EC_50_ in 10 mM phosphate buffer. We performed these low-salt experiments using vancomycin as a control for Gram-positive bacteria and polymyxin B as a control for Gram-negative bacteria. Polymyxin B was found to be very effective against all strains tested, with all EC50 values being under 1 μg/ml, except against *E. coli* ATCC 4157 (2.50 μg/ml). In the case of vancomycin, we found that this antibiotic did not kill either *S. aureus* strain under 100 μg/ml, though the MIC for both strains was 1 μg/ml, indicating that vancomycin is bacteriostatic but not reliably bactericidal.

**Table 4:** Antimicrobial activity and statistical data

Peptide	Bacteria	MIC (μg/ml)	EC50 (μg/ml)	95 % CI (μg/ml)	EC50 (μM)
Apo5	*E. coli* ATCC 4157	NT	19.7	13.1 to 29.6	6.35
	*E. coli* ATCC 51659	>250	13.9	10.7 to 18.0	4.48
	*S. aureus* ATCC BAA-1718	NT	4.96	2.82 to 8.72	1.60
	*S. aureus* ATCC 33592	>250	0.0680	0.124 to 0.357	0.0219
	*P. aeruginosa* PAO1	>250	0.0878	0.0326 to 0.237	0.0283
	*P. aeruginosa* ATCC BAA-2110	NT	0.467	0.234 to 0.934	0.150
	*A. baumannii* ATCC 9955	NT	0.644	0.480 to 0.865	0.207
	*A. baumannii* ATCC BAA-1794	>250	0.234	0.122 to 0.450	0.0755
Apo6	*E. coli* ATCC 4157	NT	3.85	2.71 to 5.48	1.39
	*E. coli* ATCC 51659	>250	9.07	7.11 to 11.6	3.28
	*S. aureus* ATCC BAA-1718	NT	2.31	1.69 to 3.16	0.835
	*S. aureus* ATCC 33592	>250	0.883	0.526 to 1.48	0.319
	*P. aeruginosa* PAO1	>250	1.17	0.866 to 1.62	0.429
	*P. aeruginosa* ATCC BAA-2110	NT	0.130	0.100 to 0.168	0.0470
	*A. baumannii* ATCC 9955	NT	0.233	0.129 to 0.419	0.0842
	*A. baumannii* ATCC BAA-1794	>250	0.126	0.0899 to 0.176	0.126
A1P	*E. coli* ATCC 4157	NT	9.2	5.90 to 14.4	2.24
	*E. coli* ATCC 51659	>250	2.51	1.55 to 4.08	0.611
	*S. aureus* ATCC BAA-1718	NT	36.5	25.1 to 53.1	8.89
	*S. aureus* ATCC 33592	>250	2.68	1.51 to 4.76	0.653
	*P. aeruginosa* PAO1	>250	38.6	4.01 to 372	9.4
	*P. aeruginosa* ATCC BAA-2110	NT	>800		>195
	*A. baumannii* ATCC 9955	NT	24.0	1.52 to 7.19	5.84
	*A. baumannii* ATCC BAA-1794	>250	2.36	0.370 to 1.75	0.575
LL-37	*E. coli* ATCC 4157	NT	0.191	0.109 to 0.337	0.0425
	*E. coli* ATCC 51659	>250	0.298	0.208 to 0.428	0.0663
	*S. aureus* ATCC BAA-1718	NT	0.839	0.497 to 1.42	0.187
	*S. aureus* ATCC 33592	>250	0.208	0.138 to 0.312	0.0462
	*P. aeruginosa* PAO1	>250	0.647	0.598 to 6.96	0.144
	*P. aeruginosa* ATCC BAA-2110	NT	2.26	1.50 to 3.41	0.503
	*A. baumannii* ATCC 9955	NT	1.46	1.04 to 2.07	0.825
	*A. baumannii* ATCC BAA-1794	>250	0.804	0.370 to 1.75	0.179
Polymyxin B	*E. coli* ATCC 4157	NT	2.50	0.831 to 7.50	1.92
	*E. coli* ATCC 51659	NT	0.00181	0.00135 to 0.00244	0.00139
	*P. aeruginosa* PAO1	NT	0.00568	0.00458 to 0.00704	0.00436
	*P. aeruginosa* ATCC BAA-2110	NT	0.0109	0.00706 to 0.0157	0.00837
	*A. baumannii* ATCC 9955	NT	0.121	0.0895 to 0.164	0.0013
	*A. baumannii* ATCC BAA-1794	NT	0.0131	0.0111 to 0.0153	0.0101
Vancomycin	*S. aureus* ATCC BAA-1718	1	>100		>69.0
	*S. aureus* ATCC 33592	1	>100		>69.0

MIC is determined in Mueller Hinton Broth II. EC50 is determined in 10 mM sodium phosphate buffer. *NT* Not Tested

In general, it was found that the EC_50_ values of Apo5 and Apo6 were statistically similar and showed broad-spectrum activity. We found that both apolipoprotein-derived peptides had strong activity against a clinical isolate of *S. aureus* ATCC BAA-1718 (EC_50_ < 5 μg/ml). Both Apo5 and Apo6 were found to have very strong activity (EC_50_ < 1 μg/ml) against drug sensitive and MDR *A. baumannii* (ATCC BAA-1794)*,* MRSA (ATCC 33592), and MDR *P. aeruginosa* (ATCC BAA-2110). It was also found that Apo5 and Apo6 were somewhat less active against both strains of *E. coli* tested, with EC_50_ values ranging from 4 to 20 μg/ml, though our previous work demonstrated that these peptides were extremely effective against *E. coli* ATCC 25922 [8]. Apo5 and Apo6 had differing activities against *P. aeruginosa* PAO1; Apo5 was found to have stronger activity against this strain than Apo6 (0.0878 μg/ml vs 1.17 μg/ml).

A1P was found to have stronger broad-spectrum activity than anticipated from our previous study [8]. Although A1P had weak activity against *P. aeruginosa* PAO1 (EC_50_ = 38.6 μg/ml), it was not effective at concentrations tested against MDR *P. aeruginosa* (ATCC BAA-2110) (Table 4). A1P had stronger antimicrobial activity against MDR strains of *E. coli* (ATCC 51659)*, S. aureus* (ATCC 33592), and *A. baumannii* (ATCC BAA-1794)*,* with EC_50_ values between 2 and 3 μg/ml, than against the antibiotic sensitive strains tested, which had EC_50_ values ranging from 9 to 36 μg/ml.

### Membrane permeabilization and depolarization by peptides

To determine whether Apo5, Apo6, and A1P interacted with the bacterial membrane, each peptide’s ability to disrupt or permeabilize the membrane was measured by the ethidium bromide uptake assay, while membrane depolarization was measured with the fluorescent chemical DiSC_3_(5), which is sensitive to the polarization of membranes.

**Fig. 5:**
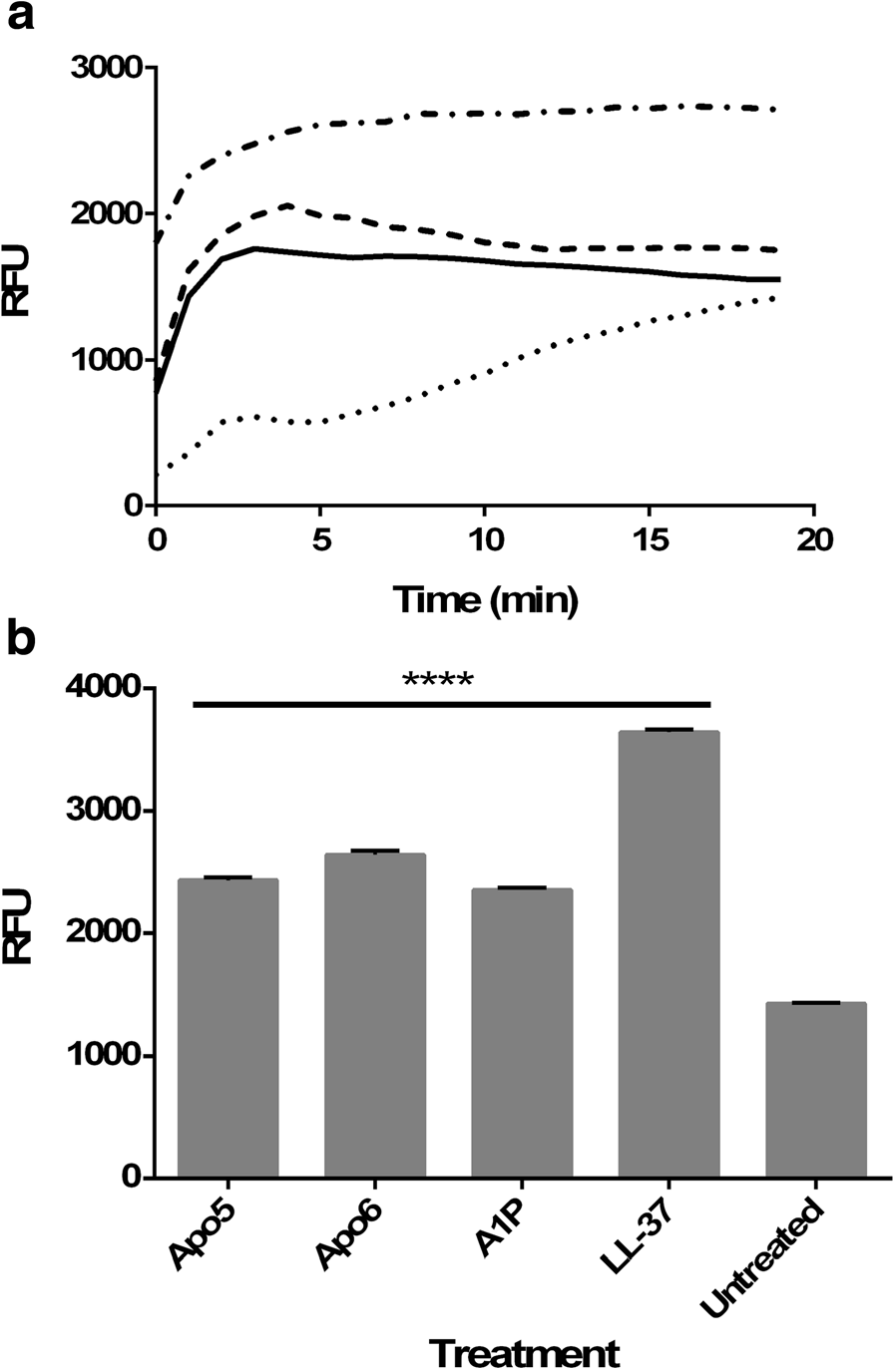
Pore-forming activity by Apo5, Apo6, and A1P. An increase in fluorescence demonstrates greater binding of DNA by ethidium bromide, which indicates the formation of pores in the bacterial membrane. **a**. Kinetics of permeabilization and binding of ethidium bromide to DNA after treatment with Apo5 (*dashes*), Apo6 (*solid line*), A1P (*dots*), and LL-37 (*dash dot*) **b**. Permeabilization of membrane after 20 min

When the ethidium bromide uptake assay was performed (Fig. 5a), it was found that Apo5 and Apo6 permeabilized the *E. coli* membrane at concentrations at 50 μg/ml quickly, comparable to control peptide LL-37, a known pore-forming peptide. Neither Apo peptide permeabilized membranes at lower concentrations. Like LL-37, these peptides permeabilized the membrane in a significant manner (*p* < 0.001), with peak fluorescence occurring within 3 min. A1P also permeabilized the membrane at 50 μg/ml significantly higher than the untreated control (*p* < 0.001), but displayed very different and slower kinetics. With this peptide, fluorescence gradually increased over the 20 min experimental time frame, until reaching nearly equivalent maximum fluorescence as Apo5 and Apo6 by the end of the experiment (Fig. 5b).

**Fig. 6:**
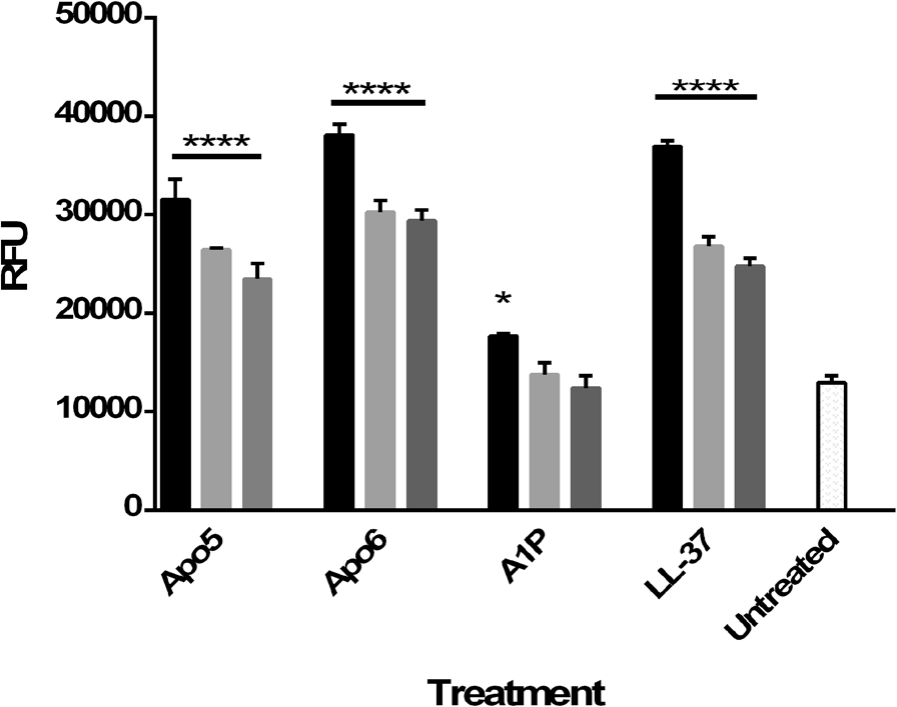
Membrane depolarization activity by Apo5, Apo6, and A1P. Depolarization determined using DiSC_3_(5) for each peptide at 50 μg/ml (■), 5 μg/ml (), 0.5 μg/ml (), as well as no treatment ()

Depolarization of a bacterial membrane indicates transient membrane disruption that allows for ion leakage, which damages the proton motive force and other gradients that store chemical energy. As shown in Fig. 6, within 1 min it was found that Apo5 and Apo6 depolarized bacterial membranes at concentrations as low as 0.5 μg/ml (p < 0.001), with depolarization showing a clear dose-dependent response to peptide concentration. A1P did not depolarize membranes except at the highest concentration tested, 50 μg/ml (*p* < 0.05). Depolarization signals at this concentration were well below those achieved at the lowest concentrations used for Apo5, Apo6, and LL-37 (*p* < 0.001).

These results indicate that Apo5 and Apo6 depolarize the bacterial membrane quickly, suggesting membrane disruption is the mechanism by which these peptides kill bacteria. A1P does not depolarize the membrane, nor does it form pores quickly except at high concentrations. This implies that the primary mechanism of A1P is not related to membrane disruption.

### DNA binding

**Fig. 7:**
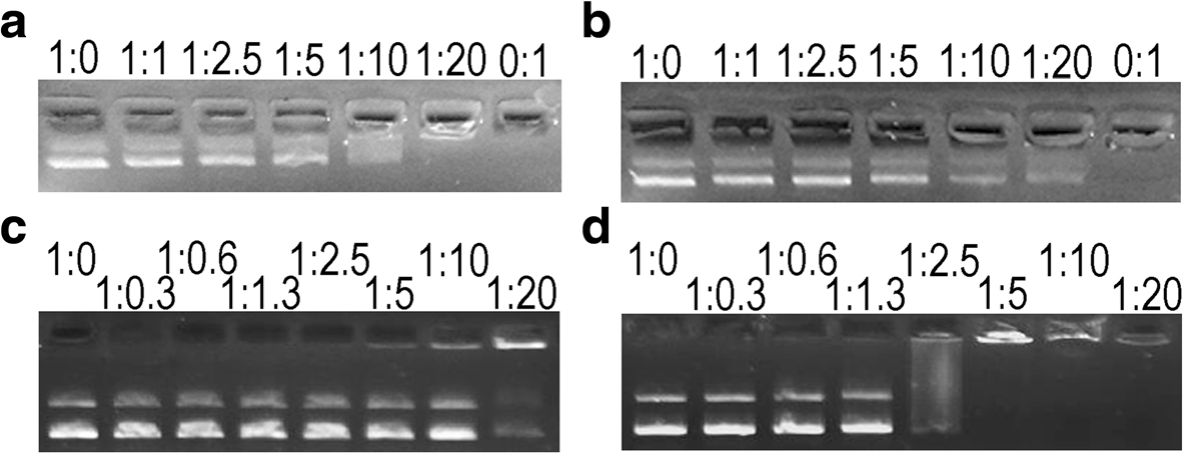
DNA binding by **a** Apo5, **b** Apo6, **c** A1P, and **d** LL-37 as measured using a gel shift assay. Ratio of DNA:CAMP is shown

Some peptides, such as LL-37 or histone-derived Buforin II, bind nucleic acids [4, 41], inhibiting translation and transcription or promoting mutagenesis. In general, this binding mechanism has been shown to be non-specific [41, 42]. To determine if any of the CAMPs were able to bind DNA, a gel shift assay was performed, shown in Fig. 7. A1P bound DNA only at very high ratios, needing at least 20 times more mass of peptide than DNA to inhibit DNA movement. Apo6 did not bind DNA at any concentration, while Apo5 bound DNA only at the highest concentration tested. It is unlikely that the primary mechanism of action of these three peptides is related to DNA binding.

### Cytotoxicity

**Fig. 8:**
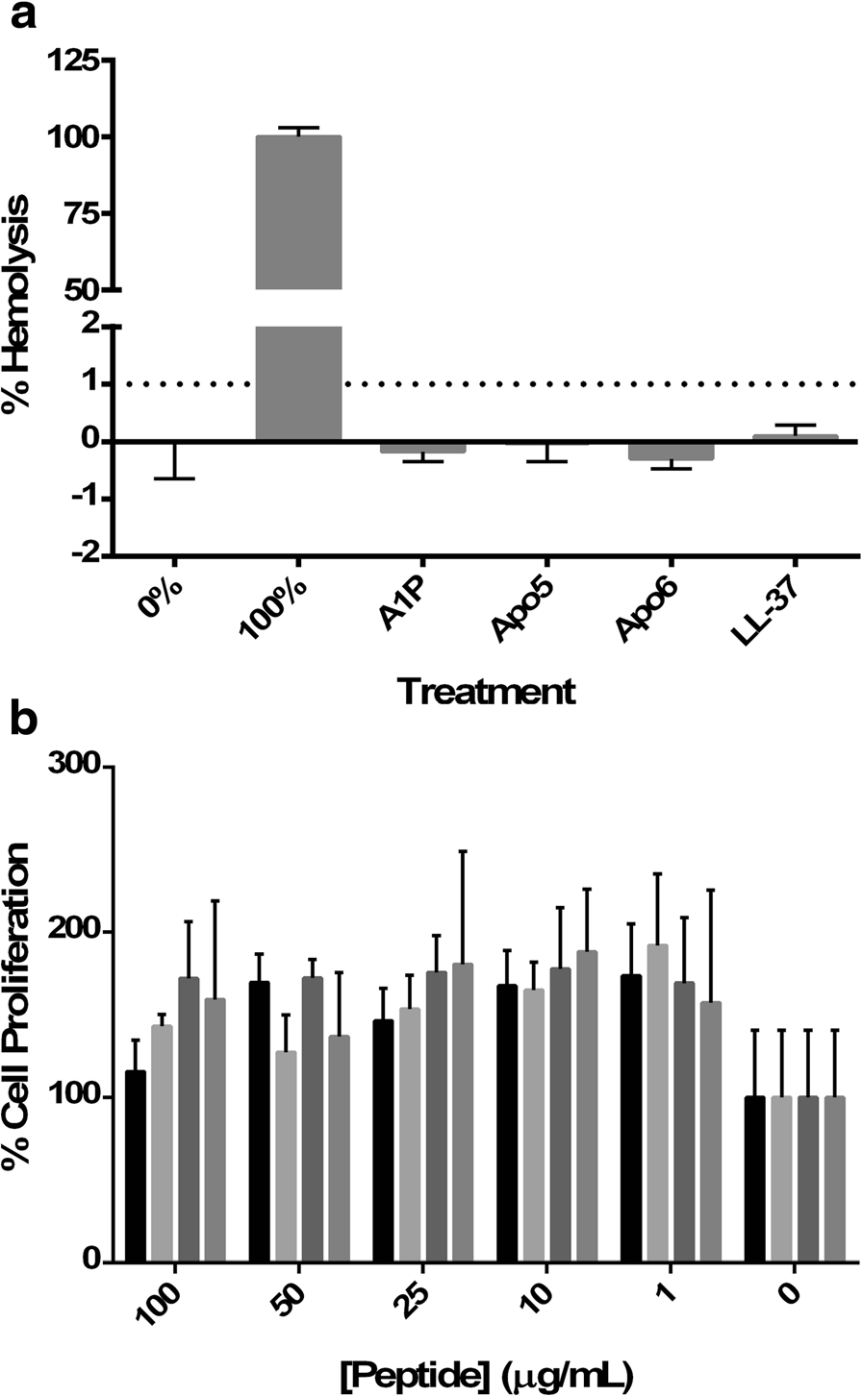
Peptide Cytotoxicity **a.** Hemolysis of sheep red blood cells was measured using a photometric assay after exposure to 300 μg/ml for 1 h and **b.** Inhibition of Proliferation of A549 human lung epithelial cells measured using the MTT assay after 24 h exposure. Apo5 (■), Apo6 (), A1P (), LL-37 ()

The physiochemical properties of CAMPs preclude significant host-cell cytotoxicity; however, some CAMPs, such as SMAP-29, have been found to cause damage to eukaryotic cells at similar concentrations. Cytotoxicity against red blood cells and A549 lung epithelial cells was measured. For red blood cells, a spectrophotometric assay that measures free heme was used, while an MTT assay was used for A549 cells. As shown in Table 4, these peptides have EC50’s between 0.07–39 μg/ml. In Fig. 8a, hemolytic activity is shown as percent hemolysis. All four peptides showed hemolysis of RBCs of less than 1 % at 300 μg/ml, a, comparable to the control peptide LL-37. No statistically significant difference was found between the untreated control and all peptides, indicating that Apo5, Apo6, and A1P are not hemolytic. The MTT assay was used to measure cytotoxicity of other cells lines. After 24 h exposure, concentrations of peptide up to 100 μg/ml were not significantly cytotoxic against A549 cells, as shown in Fig. 8b, while the EC50 against *A. baumannii* was less than 1 μg/ml of Apo peptide.

## Discussion

Using our CAMP discovery process and only 100 μl of alligator plasma, we have previously identified five novel antimicrobial peptides from *A. mississippiensis* that exhibit antibacterial activity [8]. Our bioprospecting-based process provides a unique access to the antimicrobial peptidome, and is a significant advance in the effort to identify novel antimicrobial peptides in nature. In this study, we present detailed characterization of the structure and function of three alligator plasma-derived peptides: Apo5, Apo6, and A1P. We demonstrated that Apo5, Apo6, and A1P are potent antimicrobial peptides that extend their efficacy against multi-drug resistant and clinically relevant pathogens, such as *A. baumannii.*

The two peptides Apo5 and Apo6 are both derived from a predicted apolipoprotein C-1 in *A. mississippiensis*. Apolipoproteins bind lipids; apolipoprotein C-1 in particular is known to bind phospholipids and is a marker of apoptosis [11, 12, 43, 44]. Apo6 is a smaller derivative of Apo5; Apo5 contains residues 64-88 of predicted apolipoprotein C-1, while Apo6 contains residues 67-88. These peptides were very active against Gram-negative bacteria. Previously, Apo5 and Apo6 were each found to be strongly active against *E. coli* ATCC 25922 and *P. aeruginosa* ATCC 9027 while EC_50_ values were much higher for virulent *Staphylococcus aureus* (ATCC 25923). We discovered that Apo5 and Apo6 are predominantly helical peptides. Their mode of antibacterial activity appears to be primarily through membrane interaction. At low concentrations (approximately the EC_50_ for all bacteria), Apo5 and Apo6 depolarized the membrane of *E. coli*. At high concentrations, well above the EC_50_ for all bacteria tested, these peptides more strongly disrupted the bacterial membrane, possibly via pore formation, allowing for the escape of large cellular milieu. Based on these observations, it seems that the primary mode of action of these apolipoprotein-derived peptides is membrane perturbation and depolarization.

The antimicrobial activity of apolipoproteins has been highlighted in other organisms, although typically apolipoprotein A is studied [9–12, 14, 45]. Many whole apolipoproteins have been found to have antimicrobial activity against a variety of bacteria, as well as some viruses. Apolipoprotein A-1 from various fishes have been shown to have antimicrobial activity against *E. coli* [11, 12, 46], *Streptococcus* spp. [11] and fish pathogens such as *Mycobacterium marinum* [11] and *Yersinia ruckeri* [12]. The antimicrobial activity of human apolipoprotein A-1 has also been examined, and several groups have found that this protein is effective against *E. coli, Klebsiella pneumoniae,* and *Yersinia enterocolitica* [10, 44]*.* The primary mode of action of apolipoprotein A-1 seems to be as a detergent; it has been shown to bind lipids including LPS and to dissolve micelles [12, 44, 47]. The LPS-binding activity in particular has been shown to be reliant on the N-terminus of the protein [44]. The mechanism of action and antimicrobial activity of apolipoprotein C-1 has not yet been explored, but it likely binds lipids as some part of its mechanism.

Apo5 and Apo6 were not found to bind *E. coli* LPS as part of its mechanism *(data not shown)*, but these two peptides are located on the C-terminus of apolipoprotein C-1. To our knowledge, fragments of apolipoproteins have not been characterized prior to this work. Other groups have studied synthetic mimetics of apolipoprotein E, which have been found to have anti-infective effects against HIV-1 and HSV-1 [48] and antimicrobial activity against MDR bacteria [14]. This characterization of the broad antimicrobial activity of apolipoprotein C-1-derived peptides in the present work adds to the understanding of this family of CAMPs.

A1P is a fragment of a predicted alpha-1-proteinase. A1P contains amino acids 394-428 of this proteinase inhibitor. Though the primary role of alpha-1-proteinase is the inhibition of human neutrophil elastase, it also inhibits a number of other proteases, such as proteinase 3 [49], some kallikreins [50], matriptase [51], and caspase-3 [52]. It has also been shown to have an important role in immune modulation. Alpha-1-proteinase is an acute-phase reactant; levels of this protein increase 3 to 4 fold in response to PAMPs or inflammation [53, 54]. Alpha-1-proteinase is involved in neutrophil degranulation [55]. It has also been shown to protect mice from LPS- and TNF-associated lethality, possibly by preventing the release of membrane-bound TNF [56]. Alpha-1-proteinase also interacts with human neutrophil peptides to reduce their cytotoxic affects towards lung cells; this has been demonstrated in vitro and in vivo [57]*.*

This protein may play a role in the disease progression of some bacteria such as *Francisella tularensis.* Studies have shown that alpha-1-proteinase is decreased during a pulmonary *F. tularensis* challenge in mice. This could lead to unchecked neutrophil elastase activity, which damages the alveoli and lung tissue, allowing bacteria to quickly disseminate through the body and to the liver [58]. Though other protease inhibitors have been shown to have antimicrobial activity, previously α-1-proteinase and its fragments had only been shown to have anti-infective activity, not direct antimicrobial activity. The whole protein has shown to have protective activity against *P. aeruginosa,* by suppressing bacterial proliferation and tissue inflammation [59, 60]. Whole alpha-1-proteinase, as well as a fragment and cyclic derivatives, have demonstrated the ability to prevent HIV-1 infection, possibly by inhibiting production of the virus [61–63]. Interestingly, this anti-HIV-1 fragment is the C-terminal fragment of the protein, where A1P is also found. The antibacterial mechanism of action is not yet clear from the scope of this study. A1P was not found to depolarize the bacterial membrane or form larger pores except at concentrations much higher than calculated EC_50_ values. A1P was also not found to bind DNA effectively. However, considering that A1P does interact with the membrane and form pores in high concentrations slowly, as discussed in section 3.4, it is possible that A1P crosses the bacterial membrane and interacts with a non-nucleic acid target or inhibits bacterial proteases that are necessary for viability. It seems possible that the anti-HIV-1 function described by other groups is the same function that kills bacteria.

Vancomycin and polymyxin B are drugs of last resort in the treatment of extremely multi-drug-resistant bacteria, and yet resistance to even these two drugs is currently rising [64–66]. In addition, both vancomycin and polymyxin B are known to be hemolytic and cytotoxic to some cell lines [67, 68]. It is clear that new antimicrobials are needed, particularly drugs with low cytotoxicity. LL-37 is a well-studied peptide with broad-spectrum activity. In this study, we have used LL-37 as a peptide control. We found that the alligator CAMPs tested had comparable activity to LL-37 against some species. LL-37 was found to have very strong activity against all species tested, with EC_50_ values all below 3 μg/ml. Apo5 and Apo6 had comparable activity against strains of *P. aeruginosa, A. baumannii,* and MRSA (ATCC 33592), but were less active against strains of *E. coli* tested, with EC_50_ values ranging from 2–20 μg/ml. These peptides also had similar membrane perturbation activity as LL-37, with membrane depolarization at low concentrations and pore formation and disruption at very high concentrations. A1P had weaker activity than LL-37 against susceptible strains of bacteria tested (EC_50_ values between 9 and 40 μg/ml) but stronger and comparable activity to LL-37 against drug-resistant strains of *E. coli*, *S. aureus,* and *A. baumannii*, with EC_50_ values less than3 μg/ml. Interestingly, A1P was not active against drug-resistant *P. aeruginosa*. We performed experiments with ethidium bromide and Disc_3_(5) using A1P against both strains of *P. aeruginosa* at 40 μg/ml, the approximate EC_50_ of A1P against *P. aeruginosa* PAO1, to determine if this difference in activity was due to differences in membrane interaction (data not shown). However, there was no difference in either depolarization or disruption. In our previous work, we found that A1P was least effective against *P. aeruginosa* ATCC 9027 in a panel also including other strains of *S. aureus, E. coli*, and *Bacillus cereus* [8]. It is possible that *P. aeruginosa* is intrinsically resistant to this particular peptide.

The novel CAMPs characterized here are among the first discovered using our hydrogel bioprospecting technology, illustrating the power of this technology to capture naturally occurring small peptides with net cationic charge and antimicrobial activity [8]. Considering the strong and broad-spectrum activity of these CAMPs, it seems likely that there is a greater role for protein fragments in peptide-based innate immunity than previously thought. Apolipoproteins are a highly conserved class of proteins, and it is very likely that fragments are produced in similar patterns across species. For example, though the human apolipoprotein C-I (accession AAH55093) shares 41 % identity with the American alligator version, the C-terminal 25 aa on each protein, where Apo5 is found, shares 59 % identity. This theoretical fragment of the human apolipoprotein C-I, with the sequence ELSAKMREWFSETFQKVKEKLKIDS, is also predicted to be helical, with a weaker hydrophobic moment (0.375 μH) and similar hydrophobicity (36 %), but is less charged the alligator Apo5 (+1), according to Heliquest [26] and The Antimicrobial Peptide Database [16]. The human peptide is not predicted to be antimicrobial by CAMPR3, but neither is Apo5, and we have discussed previously the weaknesses of commonly used antimicrobial peptide predictors [8]. We are currently working to determine whether some of these fragmentation patterns are conserved across species.

In addition, these CAMPs are more effective against drug-resistant strains than normal laboratory strains, indicating these CAMPs may be valuable basis for treatments against multi-drug resistant bacteria. Considering the strength of Apo5 and Apo6 on MDR *A. baumannii* in particular and the low cytotoxicity of these peptides, these are strong candidates for in vivo testing, or to use as scaffolds for stronger synthetic CAMPs.

## Conclusion

Apo5, Apo6 and A1P are antimicrobial peptides found the plasma of the American alligator. Apo5 and Apo6 are alpha-helical C-terminal fragments of apolipoprotein C-1. These peptides have strong activity against *S. aureus* and a number of Gram-negative bacteria, including MDR *A. baumannii* and *P. aeruginosa*. Apo5 and Apo6 primarily work by depolarizing the bacterial membrane. A1P is a C-terminal fragment of the alligator alpha-1-proteinase. It has strong activity against *S. aureus* and a number of Gram-negative bacteria, but not MDR *P. aeruginosa*. A1P has a mixed structure, and the mechanism of action was not clear based on experiments performed; however, it does seem to slowly disrupt the bacterial membrane.

These peptides are fragments of conserved proteins found in many animals, indicating that these peptides could play a role in immune response of many animals. In addition, new antimicrobials are desperately needed against MDR Gram-negative bacteria. Apo5 and Apo6 in particular have strong activity against MDR pathogens of clinical interest, and the native peptides or synthetic variations will make strong candidates for in vivo testing and pre-clinical trials.

## Abbreviations

ATCC, American type culture collection; BLAST, basic local alignment search tool; BSA, bovine serum albumin; CAMP, cationic antimicrobial peptide; CD, circular dichroism; CI, 95 % confidence intervals; DiSC3(5), 3,3’- dipropylthiadicarbocyanine iodide; DTT, dithiothreitol; EC50, half maximal effective concentration; EDTA, ethylenediaminetetraacetic acid; ESI-MS, electrospray ionization mass spectrometry; Fmoc, fluorenylmethyloxycarbonyl; HIV, human immunodeficiency virus; HPLC, high performance liquid chromatography; LPS, lipopolysaccharide; MDR, multi-drug resistant; MTT, 3-(4,5-Dimethylthiazol-2-yl)-2,5-diphenyltetrazolium bromide; NCBI, National Center for Biotechnology Information; OD, optical density; PAMP, pathogen-associated molecular pattern; PBS, phosphate buffered saline; RBC, red blood cell; RFU, relative fluorescence units; SDS, sodium dodecyl sulfate; TFE, trifluoroethanol; TNF, tumor necrosis factor
